# Breaking barriers in trauma research: A narrative review of opportunities to leverage veterinary trauma for accelerated translation to clinical solutions for pets and people

**DOI:** 10.1017/cts.2024.513

**Published:** 2024-04-05

**Authors:** Kelly E. Hall, Claire Tucker, Julie A. Dunn, Tracy Webb, Sarah A. Watts, Emrys Kirkman, Julien Guillaumin, Guillaume L. Hoareau, Heather F. Pidcoke

**Affiliations:** 1 Department of Clinical Sciences, College of Veterinary Medicine & Biomedical Sciences, Colorado State University, Fort Collins, CO, USA; 2 Translational Trauma Research Alliance (TeTRA-Med), Fort Collins, CO, USA; 3 One Health Institute, Office of the Vice President of Research and Department of Clinical Sciences, College of Veterinary Medicine & Biomedical Sciences, Colorado State University, Fort Collins, CO, USA; 4 Medical Center of the Rockies, University of Colorado Health North, Loveland, CO, USA; 5 CBR Division, Medical and Trauma Sciences Porton Down, Salisbury, WI, UK; 6 CBR Division, Dstl Porton Down, Salisbury, WI, UK; 7 Emergency Medicine Department and Nora Eccles-Harrison Cardiovascular Research and Training Institute and Biomedical Engineering Department, University of Utah, Salt Lake City, UT, USA

**Keywords:** Austere care, models, one health, one medicine, systems biology, traumatic hemorrhage, traumatic brain injury

## Abstract

Trauma is a common cause of morbidity and mortality in humans and companion animals. Recent efforts in procedural development, training, quality systems, data collection, and research have positively impacted patient outcomes; however, significant unmet need still exists. Coordinated efforts by collaborative, translational, multidisciplinary teams to advance trauma care and improve outcomes have the potential to benefit both human and veterinary patient populations. Strategic use of veterinary clinical trials informed by expertise along the research spectrum (i.e., benchtop discovery, applied science and engineering, large laboratory animal models, clinical veterinary studies, and human randomized trials) can lead to increased therapeutic options for animals while accelerating and enhancing translation by providing early data to reduce the cost and the risk of failed human clinical trials. Active topics of collaboration across the translational continuum include advancements in resuscitation (including austere environments), acute traumatic coagulopathy, trauma-induced coagulopathy, traumatic brain injury, systems biology, and trauma immunology. Mechanisms to improve funding and support innovative team science approaches to current problems in trauma care can accelerate needed, sustainable, and impactful progress in the field. This review article summarizes our current understanding of veterinary and human trauma, thereby identifying knowledge gaps and opportunities for collaborative, translational research to improve multispecies outcomes. This translational trauma group of MDs, PhDs, and DVMs posit that a common understanding of injury patterns and resulting cellular dysregulation in humans and companion animals has the potential to accelerate translation of research findings into clinical solutions.

## Introduction

Trauma can affect any individual and accounts for approximately 6 million deaths per year globally, representing ∼ 10% of human mortality/year [1]. In the USA alone, injuries cost $4.2 trillion USD in a single year and are responsible for the most life-years-lost in people younger than 70 [2]. Household pets, such as dogs and cats (i.e., companion animals), also experience significant morbidity and mortality due to traumatic injury, which is the second leading cause of death in pet dogs in the USA and the leading cause of death in pet cats in the United Kingdom [3,4]. Preclinical studies (induced models) have significant advantages, such as standardization, controlled environments, and ability to manipulate injury severity, and they are critical for understanding mechanisms and sequelae of injury. Unfortunately, a high percentage of preclinical studies fail to translate into successful Phase I and II human clinical trials [5]. Morbidity and mortality due to unmet medical needs as patients await effective solutions are significant. The military has spent>50 years seeking approval of cryopreserved platelets for life-saving damage control resuscitation; they have yet to be FDA approved for human use, and the cost in potential lives saved is difficult to assess [6,7]. Of the top 5 causes of life-years-lost in the USA, trauma is the highest but receives a fraction of the available funding [2]. Clinical pet (companion) animal studies, even in well-funded areas like oncology, are further cost-constrained as the bulk of federal funding goes to preclinical and human clinical research [8].

It currently takes an average of 15 years and $1-2 billion to achieve marketing approval for a new drug in the US pharmaceutical industry, and device and diagnostics sectors statistics are similarly escalating [9]. Overall, only ∼ 10% of drug candidates achieve regulatory approval due to complications and inefficiencies in development and approval pathways [5]. Only one of ∼ 10,000 promising compounds typically produces a US Food and Drug Administration (FDA)–approved treatment. Two causes identified for this failure are lack of clinical efficacy and unmanageable toxicity or side effects, with poor pharmacokinetics and poor strategic planning also playing a role [5]. Preclinical models lack the exposures, comorbidities, and trained immune responses seen in pets and humans, which can impact treatment outcomes. The resultant loss in time, resources, and effective therapies are costly for development programs and patients alike. One potential solution is to fund veterinary patient research, termed “naturally occurring models of injury,” when they replicate trauma responses exhibited by injured humans.

Naturally occurring companion animal (i.e., pet) models have successfully served as a bridge between preclinical studies and human clinical trials, delivering important observations that contributed to pre-phase I and II go/no-go decisions. For example, genetic similarities in human and canine osteosarcoma enabled rapid testing and drug repurposing in pet dogs, accelerating the discovery of successful interventions translated to therapies for people [10] Similarly, the use of veterinary patients as a proof-of-concept for human cell therapies has been discussed [11,12]. Another benefit is the ability to address confounders prior to moving to human trials. The value of these studies is found in the lifestyle and exposures of companion animals. Like humans, they have similar genetics, comorbidities, microbial and vaccination exposures, unhealthy lifestyles, and are subject to inconsistent application of therapies due to non-compliance and missed doses [8]. Also, like their human counterparts, companion animals can be treated in well-resourced veterinary hospitals where clinical trials closely mimic Phase I-III clinical trials but with less regulatory and financial burden (Supplement 2). Well-informed companion animal (pet) studies offer the opportunity to improve the product development pathway to the benefit of both human and nonhuman animal trauma patients. This approach to improving the safety, reducing the cost, and increasing the probability of success of human clinical trials is being explored in the fields of oncology, neurology, infectious disease, and cognitive dysfunction associated with aging, to name a few [13–16]. In trauma research, there are no published studies that quantify the financial, trial design, or clinical benefits of studying interventions in veterinary patients prior to moving to human subjects because the strategy is in its infancy.

This narrative review article summarizes our current understanding of veterinary and human trauma, thereby identifying knowledge gaps and opportunities for collaborative, translational research to improve multispecies outcomes. This translational trauma group of MDs, PhDs, and DVMs theorizes that a common understanding of injury patterns and resulting cellular dysregulation in humans and companion animals has the potential to accelerate the translation of research findings into clinical solutions [17]. Further, the author group believes there is an opportunity to leverage naturally occurring trauma in companion animals (i.e., pets sustaining injury) as a model to identify trauma-focused clinical solutions more rapidly [18,19]. The purpose of this review is to outline the evidence supporting the hypothesis that veterinary studies have a potential role and could provide benefits to both human and veterinary patients. Such an approach requires a detailed knowledge of the similarities and differences in the physiology, injury patterns, and treatment environment in which pets receive trauma care, in order to facilitate correct interpretation of study results. We have chosen to focus on current resources and knowledge to support this approach (section I), trauma-related syndromes that result in high morbidity and mortality in humans and pets alike (section II: traumatic hemorrhage, trauma-induced coagulopathy, traumatic brain injury (TBI)), and emergent topics (section III: systems biology, trauma immunology).

## Resources and discovery environment

### Canine preclinical models

Purpose-bred (i.e., laboratory) dogs have been used in translational research. Canine models of traumatic injuries have stimulated advances and changes in clinical practice [20,21]. While cell- and rodent-based research is crucial for understanding mechanisms of disease and therapeutic discovery, large animal (e.g., dogs, pigs, and sheep) research allows additional steps toward clinical translation. However, societal and ethical issues limit the use of the purpose-bred dogs (i.e., laboratory) in translational research despite the importance of laboratory-based large animal preclinical models. A large animal model of spontaneous trauma – also termed a natural animal model - would overcome those limitations, providing treatment for the injured pet dog and better replicating real-life injuries and responses. By performing research in clinical veterinary patients, researchers also obtain results in a more genetically diverse canine population.

The utility of preclinical (i.e., induced) dog models to study a wide range of complex traumatic injuries is well recognized and supports the idea that natural canine models of trauma could provide important observations and improve translation from preclinical small animal models to human subjects. Preclinical dog models have provided valuable contributions to the available research in TBI [22], hemorrhagic shock [23], and musculoskeletal injuries [24], among others, and allow intensive care level monitoring using commercially available ventilatory support and cardiac monitoring equipment, which is challenging to implement in rodents. Examples relating to TBI include using purpose-bred dogs to study new implant material for promoting neuroregeneration and improving motor function recovery after TBI [25]. 3D-printed implants loaded with hypoxia-induced exosomes promoted neuroregeneration and angiogenesis, inhibited nerve cell apoptosis and proinflammatory factor expression, and ultimately enhanced functional motor recovery in dogs subjected to TBI [25]. Another study showed the potential of 3D-printed collagen/chitosan/secretome derived from human umbilical cord blood mesenchymal stem cell scaffolds as a therapeutic option for TBI in dogs [26]. This study also outlines the potential for using dogs in neurobehavioral testing due to their complex behaviors.

Translatable models are essential to develop targeted treatment strategies for traumatic injuries. The need to further develop translational modeling is presented throughout the trauma literature citing the urgency of identifying new animal models that include a variety of species and reflect the natural clinical trajectory of trauma patients [20,27]. This relative deficiency highlights the need to develop valid and reproducible animal trauma models further. Well-designed models will facilitate improved mechanistic understanding and the development of targeted treatment strategies for traumatic coagulopathy. While there are significant advantages to working with dogs over smaller laboratory animals, the limitations of controlled research environments, ethical considerations, and societal discomfort with the use of dogs as laboratory animals are a barrier [28–30]. Opportunity exists for enrolling dogs that present to veterinary trauma centers (VTCs) for treatment in research studies, thereby providing benefits for the pet dog and owner, as well as contributing valuable data for clinical development programs.

### Facilities and resources for treatment and clinical research

Trauma care and research in human patients is facilitated by a mature network of trauma systems that grew out of military and civilian collaborations and the seminal National Research Council-National Academy of Sciences (NRC-NAC) 1966 Accidental Death and Disability Report, culminating in the Optimal Resources Manual for Trauma Centers [31]. Today, resources include local, regional, national, and global trauma registries; globally recognized continuing education [Advanced Trauma Life Support (ATLS), Prehospital Trauma Life Support (PHTLS), Rural Trauma Team Development Course]; advances in research design (e.g., pragmatic, adaptive); and a host of professional organizations. Benefits include continuous improvement, well-established national and international standards, and robust exchange of ideas and knowledge (e.g., ACS-COT, AAST, ATS, Western Trauma Association, ESTES, and IATSIC). Level 1 trauma centers reflect the breadth and depth of expertise and coordination, maintaining the highest standards of excellence in clinical practice and research. Outcomes are significantly better and attributed not just to the volume of cases but also to available resources and the highly trained care and research teams [32,33].

Coordinated efforts to improve trauma resources are more recent in veterinary care but are rapidly maturing. VetCOT was officially recognized by the American College of Veterinary Emergency and Critical Care (ACVECC) in 2012, and the initial cohort of VTCs was launched with the first edition of “Resources for the Optimal Care of the Injured Veterinary Trauma Patient”[34,35]. VTC Level (I, II, and III) is determined based on available resources, evidence of trauma registry data entry, and implementation of a Performance Improvement and Patient Safety (PIPS) program (Supplement 2) [35,36].

The VTC network has the organizational structure and team expertise to partner with Level 1 human trauma centers and execute translational multicenter prospective observational studies aimed at accelerating solutions for human and veterinary patients [35]. Comparison of human and veterinary biological samples (e.g., plasma, tissue, and stool) further increases translational potential. Veterinary versions of ATLS and PHTLS courses are currently in development. These courses, co-branded by the American College of Surgeons, will be offered to veterinary primary care providers to ensure competency and confidence in stabilizing veterinary trauma patients in a variety of environments (e.g., rural, under-resourced). In states with favorable laws, these courses will train EMS/first responders to provide basic stabilization of injured animals on scene [37,38].

### Human and companion animal trauma registries and epidemiology driving discovery

In the early 1970s, a nascent trauma registry developed at Cook County Hospital was adopted and refined by the American College of Surgeons Committee on Trauma [39]. The resulting Major Trauma Outcomes Study led to the development of the American College of Surgeons National Trauma Data Bank (NTDB) and the Trauma Quality Improvement Program, both of which engendered advances from prehospital care to resuscitation practices, surgical innovation, and trauma rehabilitation [40]. In the early 2000s, the US military added the Joint Theatre Trauma System, which would eventually become the Department of Defense Trauma Registry (DoDTR) [41,42]. The lessons learned would lead to profound changes in the treatment of human trauma as physicians better understood outcomes associated with their therapeutic choices and developed clinical practice guidelines aimed at improving care.

Veterinary trauma followed suit in 2013; a product of the VTC network, the Veterinary Committee on Trauma (VetCOT) trauma registry has progressed rapidly, with more than 65,000 cases as of December 2023 [43]. Like the NTDB, the VetCOT trauma registry provides insights into veterinary injuries and is a powerful source of preliminary data for sample size calculation and recruitment justification in clinical research. The growing registry has already provided guidance aimed at improving veterinary patient outcomes[44–46]. To date, over 25 VetCOT trauma registry publications have added invaluable insights into trauma etiologies and provided validated scoring systems [47]. In 2022, the Department of Defense Military Working Dog Trauma Registry was launched in part due to a civilian-military working dog project leveraging the VetCOT trauma registry [48,49]. The VetCOT trauma registry will enhance our understanding of veterinary trauma epidemiology, providing data on TBI, geriatric trauma, sex-related outcomes, austere care for the severely injured trauma patient, and resuscitation of the acutely hemorrhaging patient [50].

Validated injury severity scores are useful to decrease bias and confounders in research, and they can supplement clinical judgment with objective measures [51]. Many well-known examples exist in human medicine. In veterinary medicine, the Animal Trauma Triage score (ATT), a measure of injury severity in dog and cat trauma patients, was proposed in a single-center population and has subsequently been validated through data from the multicenter VetCOT trauma registry [45,52]. Leveraging the VetCOT registry, a more parsimonious injury severity score with superior calibration to the ATT has been developed (VetCOT score), and a veterinary Abbreviated Injury Scale (V-AIS) is in development based on the Abbreviated Injury Score, which is focused on injury pattern, not just severity [44]. Another essential tool with translational potential is the Modified Glasgow Coma Scale (MGCS), a validated brain injury severity score in dogs and cats based on the Glasgow Coma Scale (GCS) in humans. Further research is needed to validate whether human and veterinary injury severity scores predict similar syndromes.

**Figure 1.**
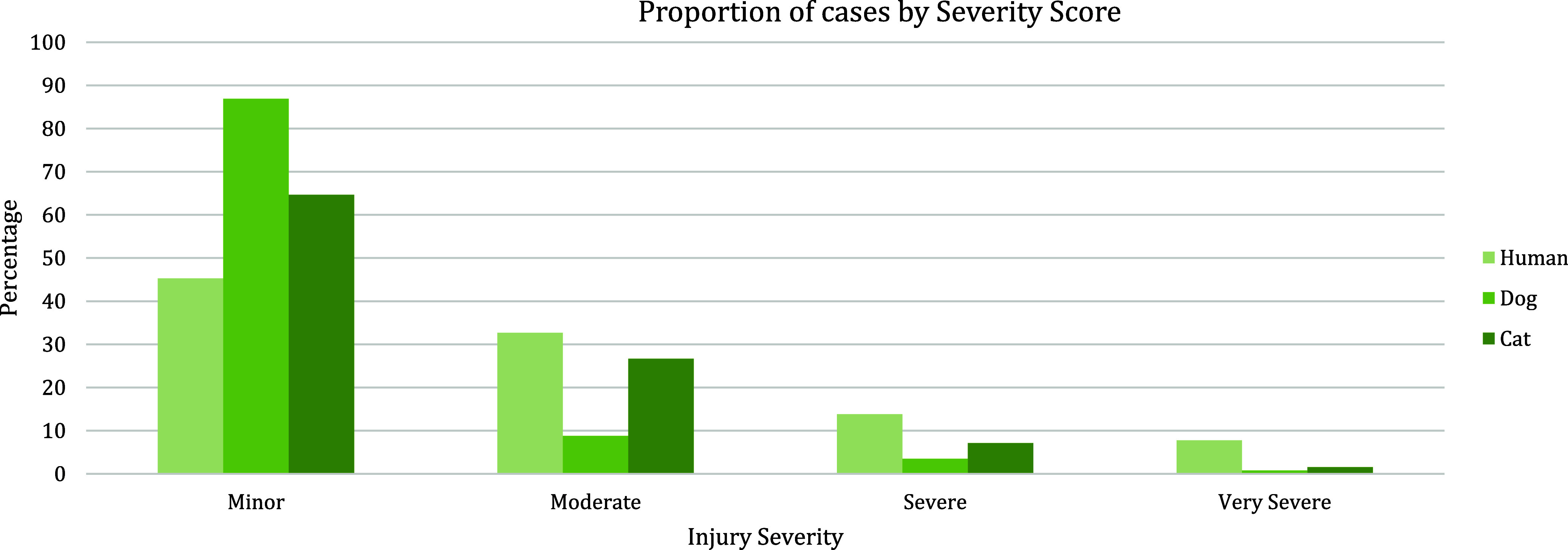
Proportion of cases by degree of injury severity in humans, dogs, and cats. Data summarized from the 2016 National Trauma Database (NTDB) and 2017–2019 Veterinary Committee on Trauma (VetCOT) registry report. Note that the VetCOT data represent data primarily from Level I and II Veterinary Trauma Centers (VTCs).

**Figure 2.**
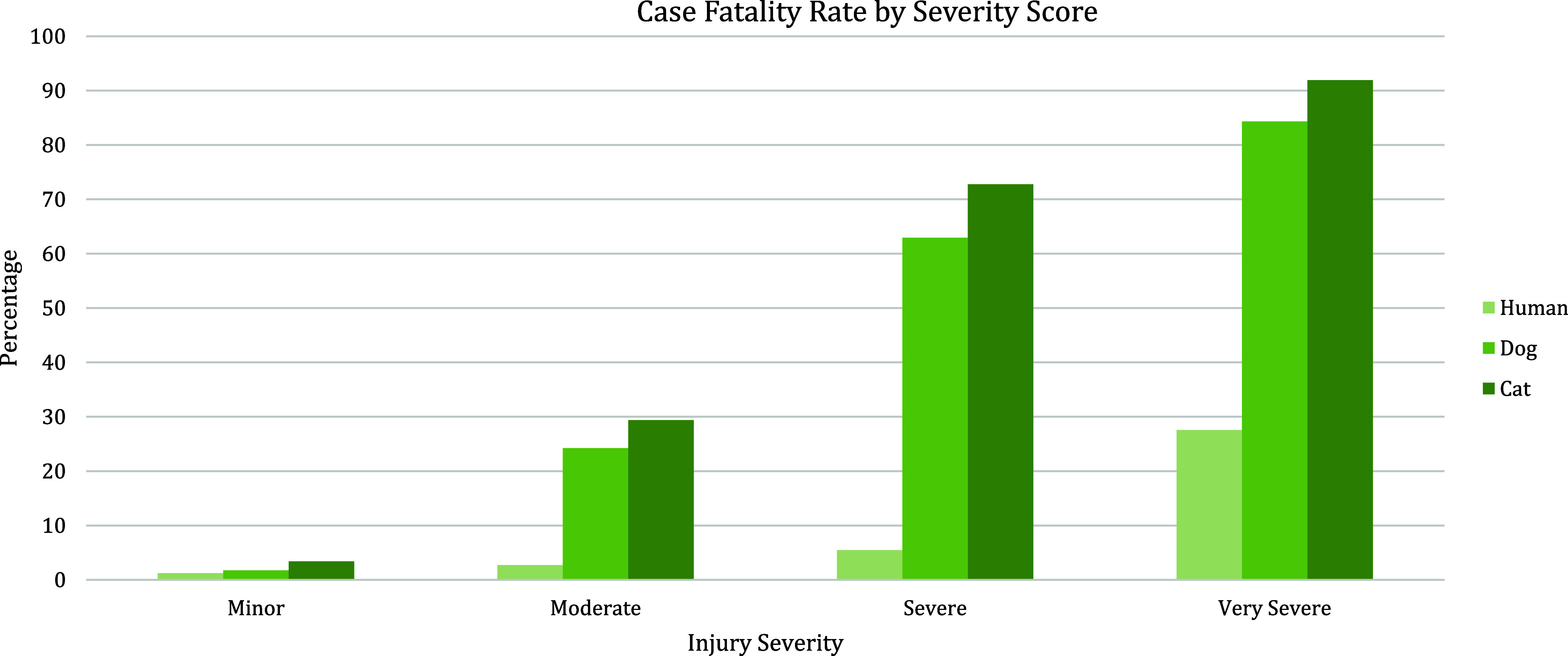
Case fatality rate by injury severity score in humans, dogs, and cats. Data summarized from the 2016 National Trauma Database (NTDB) and 2017–2019 Veterinary Committee on Trauma (VetCOT) registry report. Note that nonsurvival (fatality) in dogs and cats includes animals that are euthanized.

Some of the strengths and limitations of natural trauma models can be assessed by comparing injury mechanisms, severity, and fatality across age groups in human and veterinary trauma patients (Tables 1–3, Figs. 1 and 2). Similarities include overrepresentation of injury in younger patients, higher mortality rates in older patients, and parallels in mechanism-related fatality proportions (e.g., firearm, suffocation, fall, and vehicular injury) (Table 1) [53–55]. Table 2 highlights the overrepresentation of young adults, prominent in dog and cat populations. In the senior population, mortality rates are higher, and comorbidities are common across species (e.g., diabetes, renal disease, and hypertension). To assist evaluation of Figures 1 and 2, Table 3 divides the categories for the respective species severity scoring systems (ISS, ATT) into minor, moderate, severe, and very severe. One potential confounder of the distribution in the severity of injuries seen in Figure 1 could be due to differences in prehospital systems between the veterinary and human medical fields. In other words, more severely injured dogs and cats may die at the point of injury or be euthanized at their primary veterinary clinic prior to transport to a definitive care facility (veterinary trauma center). While case fatality rates increase with the severity of injury, as expected, the more considerable jump in dogs and cats may be related to euthanasia as an option in veterinary medicine. The impact of size (relatively uniform in cats, broad range in dogs), age, and sex on clinical course is under investigation. It is beyond the scope of this paper to adequately review the large volume of human trauma epidemiological data, but as the veterinary trauma registry continues to grow, cross-species comparisons will enhance translational insights.

**Table 1.**
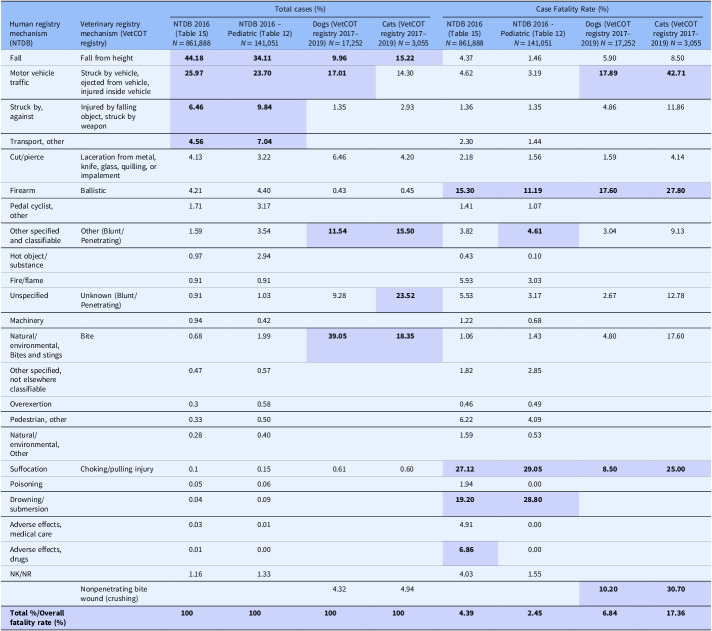
Comparison of incidence and case fatality by mechanism of injury in humans, dogs, and cats reported in the 2016 National Trauma Database (NTDB) and the 2017–2019 Veterinary Committee on Trauma (VetCOT) registry report. VetCOT mechanism categories are aligned by the authors with mechanism categories taken directly from the NTDB

*Note: The top 4 injury types and fatality by injury type are bolded and highlighted.

**Table 2.** Comparison of incidence proportion and case fatality by age in humans, dogs, and cats reported in the 2016 National Trauma Database (NTDB) and 2017–2019 Veterinary Committee on Trauma (VetCOT) registry report

AGE (years)			Proportion of total cases (%)			Case fatality rate (%)		
Category	Human	Dog/cat	Human	Dog	Cat	Human	Dog	Cat
Pediatric	<1	<1	1.08	6.92	12.34	2.19	6.73	25.20
	1-4		2.87			2.18		
	5-9		3.26			2.20		
	10-14		3.26			1.99		
Young adult	15-19	1	5.90	15.68	19.04	3.03	5.06	13.04
	20-24	2	7.67	11.00	13.04	3.88	6.46	14.81
Adult	25-34	3	12.5	9.20	8.45	3.57	5.43	19.29
	35-44	4	9.60	7.57	6.07	3.35	5.65	16.53
	45-54	5	11.28	7.24	6.67	3.42	6.04	19.92
		6		6.44	4.74		5.52	15.34
Senior	55-64	7	11.81	6.34	4.64	3.99	5.38	9.19
	65-74	8	10.23	5.88	3.64	4.84	5.22	8.28
	75-84	9	10.55	5.22	3.16	6.66	6.26	9.52
	>84	>10	9.97	18.50	18.52	8.63	10.46	20.38
Overall						**4.39**	**6.84**	**17.36**

**Table 3.** Categorization of degree of injury based on respective species injury scoring system*

	Minor	Moderate	Severe	Very severe
Human (ISS)	1–8	9–15	16–24	>24
Dog (ATT)	0–3	4–6	7–11	>11
Cat (ATT)	0–2	3–6	7–10	>10

* ISS = Injury Severity Score; ATT = Animal Trauma Triage Score.

### Barriers, differences, and opportunities in pet to human trauma translation

While there are many similarities in the mechanism and epidemiology of trauma in people and pets, it is acknowledged that differences also exist. Successful multispecies translational programs require detailed knowledge of critical inter-species differences in response to trauma and conditions of care. Supplement 1 highlights each of the trauma syndromes addressed in this article and describes advantages and disadvantages to different approaches and models in advancing trauma care.

In addition to species differences, infrastructure differences in the delivery of trauma care may impact execution of research efforts. Just as results from research conducted in low-resourced hospitals in other countries must be understood in the context of the conditions under which the clinical research was conducted, it is important to understand the similarities and differences between the standard-of-care in veterinary versus human hospitals. For example, prehospital care for human trauma is highly developed in some countries, encompassing large networks of trained first responders (EMS, EMT, paramedics) and transport systems (ground, air). Major trauma outcomes are better for those treated in Level I trauma centers, compared to those who experience long transport times, transfers from rural locations, or treatment in lower levels of care facilities [56–58]. Veterinary patients represent both groups, as some are seen at under-resourced primary veterinary clinics, and others are taken immediately to major trauma centers. Additionally, veterinary prehospital care relies mainly on transportation by pet owners and a network of primary care providers [59]. An informal transport system often results in veterinary patients initially presenting to facilities unable to address the severity of their injury. This difference creates an advantage in researching interventions and treatment under austere conditions (defined as situations where medical supplies are not enough for emergency care needs, as experienced in rural, disaster, under-resourced, or military environments). Cost constraints due to low veterinary health insurance reimbursement and perceived or real animal welfare concerns further differentiate human and veterinary care, with euthanasia as an outcome in many severely injured pets (Figs. 1 and 2). For veterinary studies that are part of a human clinical development program, these differences must be addressed in both the study design and in the interpretation of results.

While inter-species differences can hamper the translation of results, they can also provide benefits. Naturally occurring animal models of trauma can leverage the shorter average lifespans of pets to study long-term effects on a compressed timescale. The severity and duration of comorbidities between humans and pets differ and variations are important to understand, but veterinary patients develop spontaneous comorbidities such as chronic renal disease, diabetes, and obesity with many similarities to human conditions. Living alongside their human counterparts, pets are exposed to many of the same environmental, dietary, and societal health risks. Studying therapies and interventions in veterinary patients can yield important insights before translating to much more expensive human trials [8], and the long-term effects of trauma on comorbidities can be studied over years rather than decades.

## Major trauma comparisons in pets and people

### Resuscitating the hemorrhaging patient

Hemorrhagic shock is a leading cause of potentially preventable death in trauma, typically occurring within the first 3–6 hours [60,61]. This has been described in human military, military working dog, human civilian, and companion animal populations. Principles of resuscitation are well-characterized: minimize ongoing blood loss, restore oxygen delivery, prevent or treat coagulopathy, and limit endothelial glycocalyx damage. Physiologically, the response to hemorrhage between species is remarkably similar; however, the response at the cellular level may differ due to genetic, epigenetic, and health status dissimilarities.

Historically, resuscitation strategies relied primarily on crystalloid and synthetic colloids to support blood pressure until intensive research by the US military and major trauma centers definitively demonstrated that early use of hemostatic blood products is essential to prevent metabolic and endothelial derangement. In combination with early hemorrhage control, prehospital hemostatic resuscitation improves survival and prevents a host of sequelae [60,62]. Optimal ratios of hemostatic products, use of whole blood, and speed of blood product administration are areas of active research [63,64]. Details of resuscitation, including transfusion prediction parameters, endpoints of resuscitation, and adjuvant therapies such as tranexamic acid, are actively being studied in human and veterinary patients. Blood products are optimally initiated during prehospital care in human patients and military working dogs [48,62,65]. Veterinary patients treated at specialized trauma centers receive hemostatic resuscitation as early in the hospital course as possible. Regrettably, blood product availability is challenging for many veterinarians practicing in resource-limited environments, and the lack of a coordinated prehospital veterinary system can delay resuscitation and hemorrhage control. Conditions can be similar for injured people in austere settings, presenting an opportunity for translational studies. Blood products available for immediate release are the standard of care in US Level 1 human trauma centers, and they are also readily available in large non-trauma hospitals. Conversely, hemostatic products, particularly platelets, can be limited or unavailable in smaller urban, semi-rural, and rural hospitals and during the prehospital phase [66]. For many low-resource nations, conditions are far worse. In veterinary medicine, blood product availability is often limited to large specialty hospitals.

Of note, humane euthanasia and limited financial resources may reduce survival of critically bleeding animals and can be confounding for translational studies [67]. Understanding these limitations is important but does not abrogate the value of leveraging the emerging veterinary trauma system as a source of natural animal models of hemorrhagic resuscitation. In prehospital settings, humans and working dogs work shoulder to shoulder and are injured under similar conditions. Translational studies could improve outcomes for both and accelerate the path to solutions that address unmet medical needs.

### Trauma-induced coagulopathy and acute traumatic coagulopathy

When hemorrhagic shock occurs in the presence of profound tissue injury, patients can present very early with a coagulopathic syndrome known as acute traumatic coagulopathy (ATC), also described as the first phase of trauma-induced coagulopathy (TIC) [68,69]. Resuscitation-associated coagulopathy (RAC), or the second phase of TIC, is well described and results from a combination of dilution or consumption of coagulation factors, hypothermia, and acidosis as a consequence of insufficient resuscitation [70,71]. In contrast to RAC, ATC can occur acutely, is evident even during prehospital transport, and is attributable to the trauma itself. RAC is present in approximately 25% of severely injured human patients and is associated with 35–50% mortality and significant morbidity [72–74]. Both syndromes describe abnormalities in the coagulation and inflammatory systems that are associated with adverse outcomes.

Both phases of TIC contribute to early and late death in human and veterinary trauma patients [67,68,75]. In the early stages, there is prolonged bleeding and sustained hypoperfusion. In the later stages, coagulopathy may activate a systemic inflammatory cascade leading to multiple organ failure. Various coagulopathic stages develop, characterized by hypocoagulable, hyperfibrinolytic, and/or hypercoagulable phenotypes. Postulated mechanisms include activation of the protein C cascade, platelet activation, damage to the endothelium/endothelial glycocalyx, and depletion of fibrinogen [76–79]. Coagulopathy is a dynamic and complex process, and accurately defining the syndrome in people and companion animals has proved challenging [69,75,80].

In veterinary medicine, TIC has been described but is less well understood. As with people, coagulopathy increases in frequency with injury severity as measured by ATT score, hypotension, and hyperlactatemia [81]. Both hypocoagulable, hypercoagulable, and hyperfibrinolytic states are described. Fully characterizing TIC in the veterinary patient may also yield improved therapeutic strategies for humans as management appears similar for both, suggesting mechanisms may be conserved. A recent multicenter retrospective study focusing on exsanguinating dogs reported a median resuscitation ratio of 0.8 plasma: RBC; approximately 30% received an antifibrinolytic, and roughly two-thirds required surgical intervention to control critical bleeding [67].

Current TIC investigations are focused on examining the hemostatic system complexity in relation to tissue injury. Laboratory testing for coagulation abnormalities is readily available in human and veterinary medicine, including both conventional and viscoelastic testing [82–84]. However, these values often do not match the patient’s clinical coagulation status. The availability of point-of-care (POC) viscoelastic tests enables the characterization of coagulation changes after trauma as a function of time [84–86]. Coagulation laboratory tests integrated with predictive scoring systems may be the most reliable methods for early detection of TIC and guiding transfusion requirements[87]. Although prolonged clotting times predict mortality in dogs after trauma, prognosis in veterinary patients suffering from TIC is not fully described, and more descriptive studies may inform clinical research [67].

Companion animals present an excellent opportunity to expand TIC translational research in a population that more closely mimics human trauma compared to controlled laboratory experiments. Exploration along this path will help develop a more comprehensive and precise understanding of physiological and cellular responses across various clinical scenarios, with the expectation of leading to improved therapies and better patient outcomes.

### Traumatic brain injury

TBI is characterized by two phases [88]. Primary injury is the immediate and direct result of an external force to the head. Secondary injury occurs during the hours to days after trauma and is caused by a complex series of biochemical events, including the release of inflammatory mediators and excitatory neurotransmitters, and changes in cellular permeability that lead to ischemia, hypoxia, changes in blood pressure, cerebral edema, increased intracranial pressure, and hypercapnia. Treatment is directed at managing the primary injury to mitigate the impact of secondary injury.

The diagnosis, stabilization, and management of the TBI patient have many similarities between humans and animals [89,90]. Both use a baseline assessment: the GCS, used in humans, is the basis for the MGCS in companion animals [91,92]. Intracranial imaging is the standard of care in people, and while inconsistently applied in veterinary medicine due to resource constraints (cost and availability), sedated computed tomography with contrast and magnetic resonance imaging is becoming more common [93,94].

To limit injury after TBI, therapeutic interventions are directed at minimizing primary injury damage and preventing secondary injury [95]. Several animal models for TBI have been proposed, including fluid percussion injury, control cortical impact injury, weight drop impact acceleration injury, and blast injury [96]. These preclinical studies are used to test TBI interventions [96], and findings show that initiating treatment within a few hours after impact is neuroprotective in TBI animal models. Unfortunately, the translation of promising therapeutics (e.g., calcium channel blockers, osmotherapy, amantadine, and erythropoietin) proved disappointing in Phase I-III clinical human trials [97–100].

A significant difference in the management of human versus veterinary TBI is attributable to cognitive demand [101]. Companion animals have lower cognitive needs and thus are less impacted by severe TBI. Veterinary medical management is therefore based on clinical presentation and mitigating the consequences of secondary injury [90]. Working dogs and other highly trained veterinary patients may provide better TBI natural animal models, due to better discrimination of injury severity. Advanced neurorehabilitation aimed at restoring function, including attention, memory, communication, and executive function, is beneficial to humans and merits further study in veterinary trauma populations [102,103].

Unanswered questions regarding optimal human and veterinary TBI treatment are numerous. The variety of clinical injury presentations makes replication in experimental animal models complex and enrollment for clinical trials challenging [96]. Sequelae linked to other aspects of trauma – including TIC and resuscitation of the TBI patient – have yet to be fully characterized. Biomarkers to differentiate mild from moderate TBI in people are a key area of interest, as differentiating degree of severity determines the type of treating physician required [104]. The opportunity to leverage veterinary clinical studies to improve translation will depend on a comprehensive description of natural history, physiology, and cellular derangements in companion animals, leading to a better understanding of the strengths and limitations of a potential TBI natural animal model. The ultimate goal is both veterinary therapies and a less costly, more reliable transition from preclinical studies to human clinical trials.

## Emergent topics

### Translational systems biology

Mammals rely on complex biological processes dictated by genetics and translated via protein and metabolic pathways. These processes intersect with environmental factors that drive responses to threats and determine outcomes via complex systems that defy simple analyses. Immune-commanded inflammatory pathways mediate response to injury at the molecular, cellular, tissue, organ, and whole-organism levels. The advent of “omics” methodologies has supplied a wealth of data and the theoretical capability to interrogate the complete responses of cells and tissues. Combined with advanced computational techniques, this knowledge can uncover novel pathways from multidimensional data to provide mechanistic insights within and across species [105,106].

Genomics provides information on gene expression in cells or tissues at a given time. Much of the trauma-related genomic knowledge is derived from the large-scale collaborative research program entitled “Inflammation and the Host Response to Injury” [107]. Genome-wide expression analysis has been performed on circulating leukocytes obtained from adults following either severe blunt trauma or thermal injury and is often referred to as the “genomic storm” [108,109]. Early genomic profiling may serve as a highly sensitive prognostic tool for identifying trauma patients at risk of adverse outcomes and is likely age dependent [110].

Likewise, metabolomic methods quantify metabolites within biological fluids, cells, and tissues. The identities, concentrations, and fluxes of these compounds result from a complex interplay among gene expression, protein expression, and the environment. Metabolomics can supply quantitative data and identify metabolic signatures associated with conditions of interest, including drug exposure and the impact of interventions [111]. Specimens collected hours and days post-injury from both animal models of polytrauma/hemorrhagic shock and human patients have shown severe metabolic disruption [112–114]. Trauma leads to disturbances in carbohydrate, protein, and fatty acid metabolism, allowing clear discrimination between survivors and non-survivors. Metabolic profiling in the early post-injury phase may be valuable for identifying patients at an increased risk of posttraumatic complications [115]. Metabolomic and proteomic data showed accurate discrimination between human septic shock patients and those undergoing a systemic inflammatory response syndrome (SIRS) in the absence of infection [116]. Advances in artificial intelligence analyses and personalized medicine approaches may be required for the utility of Omics data in real time. Shown to perform better than statistical models built on clinical scoring systems, these data highlight the improved discriminatory power that can be gained by combining system-based approaches [116].

Considerable gaps remain in our understanding of the complex systems related to trauma immuno-inflammation. Proteomic analyses can often differentiate organism species based on protein sequence [117]. Species determination in metabolomics is challenging as small molecules are often conserved across different organisms [118]. However, this can be advantageous for animal model studies using metabolomics as knowledge of physical properties guiding identifications can be shared across species [119]. Expanding metabolomic studies to include natural animal models of injury may offer additional insight and provide an expanded database from which to derive answers to complex biological processes. Knowledge gaps include species similarities and differences in platelet/endothelial cell interactions, leukocyte activation, signaling between the nervous system and gut microbiome, and the impact of nervous system signaling on the immune response. Insights from naturally occurring animal models may be applicable to human medicine and vice versa.

Innate immune responses, inflammatory pathways, and adaptive immunity across a broad spectrum of injuries have consistently been linked to adverse outcomes, suggesting that common mechanisms likely underlie dysregulation. Given the limited number of studies and the size of the cohorts analyzed, further work is needed to validate published observations. Combining data from the naturally occurring animal model of injury and including pertinent clinical information, such as injury severity, sex, and age, can accelerate this knowledge [120]. Currently, the adequacy of resuscitation is measured using clinical signs, noninvasive measures of intravascular volume, and other endpoints of resuscitation, such as lactate and base deficit. However, these methods are too crude to understand cellular and subcellular changes that occur in trauma patients. Better diagnostic and therapeutic markers are needed to assess the adequacy of interventions, monitor responses at cellular and subcellular levels, and inform clinical decision-making prior to clinically apparent complications. The evolving field of “-omics” combined with techniques for multidimensional analyses holds great promise in the identification and application of biochemical markers to support the clinical decision-making process [121]. It is conceivable that combining these techniques can be used in the future to create tailored treatment, management protocols, and identify novel therapeutic targets.

### Trauma immunology

The intricacy and importance of the immune response to trauma have gained increased appreciation in the past few decades. Advances in early care (i.e., hemorrhage control, hemostatic transfusion, and damage control surgery) have decreased early and late trauma-related patient deaths. Delayed trauma mortality is often the interplay of complex pathophysiologic processes: the immune response to the inciting event, medical and surgical interventions, and individual patient factors. Although the understanding of these processes has improved, much remains unknown about the trauma immune response, including the identification of early biomarkers, optimal monitoring strategies, and innovative, impactful treatment options.

The desired outcome after trauma is the restoration of the preinjury state and the prevention of disordered repair mechanisms [122,123]. This requires the cessation of hemorrhage, resolution of shock, repair and/or removal of damaged tissues, prevention of infection, and re-establishment of immune homeostasis [124]. The immune response to trauma is twofold. Cellular damage causes the release of cellular and matrix components recognized as damage-associated molecular patterns, which invoke the innate immune response through pattern recognition receptors (PRRs) [123]. This subsequently results in the activation of phagocytic cells, professional antigen-presenting cells, and complement and coagulation cascades that aim to remove cellular debris, facilitate tissue repair, and translate the initial innate immune response into a longer-lasting, restorative, adaptive immune response [123].

Many factors play a role in propagating the immune response, leading to dysfunctional responses associated with increased morbidity. The combination of overwhelming trauma, secondary injuries due to surgical intervention, nosocomial infections, patient age and sex, immunocompromising comorbidities, and unfavorable epigenetic or microbiome alterations can all contribute to immune dysfunction [123]. Excessive and extensive activation of the innate immune response can cause additional tissue damage and dysfunction, which can be further exacerbated by the recognition of pathogen-associated molecular patterns from invading microbes, leading to additional, systemic activation of PRRs and inflammation [125]. Ultimately, this can lead to SIRS, which can contribute to immune cell exhaustion, immunoparesis, sepsis, and a poor prognosis. SIRS can lead to further barrier and endothelial dysfunction and early multi-organ dysfunction syndrome (MODS). Although a compensatory anti-inflammatory response syndrome occurs, it often does not overcome excessive and prolonged inflammation. Ultimately, the decreased number and functionality of immune cells can also lead to the development of late MODS and associated persistent inflammation-immunosuppressive catabolism syndrome, increasing mortality due to impaired wound healing and infection risk [123]. Elucidating the processes and details of the trauma immune response may identify markers of early immune dysfunction in at-risk patients, leading to interventions that reduce associated morbidity and mortality.

Although often limited by available funding and resources, some studies have evaluated immune responses to injury in veterinary patients. Studies in dogs with naturally occurring spinal cord injury have found post-injury immune responses that are similar to those seen in humans and rodent models [126,127], and that were correlated with injury severity, duration of injury, and post-injury outcome [126]. Some veterinary studies have evaluated components of the immune response that would be applicable to trauma patients, and the area is ripe for additional, impactful research. For example, plasma levels of acute phase proteins (APPs), which are components of the innate immune response, are altered in proportion to the severity and extent of tissue damage within hours of injury and can serve as early markers of inflammation in veterinary species [128]. Due to the promising initial studies, several APP were tested in marine mammals, for which injury identification can be challenging. However, several hurdles exist to the clinical use of APP in veterinary medicine, including the lack of specificity of APPs, knowledge gaps in APP biology and species-specific differences, and lack of species-specific reagents [128]. Although identifying current barriers, this study exemplifies the potential clinical application of increases in knowledge of the immune response to trauma for all species.

Recognition of the importance of trauma immunology is new, and knowledge gaps exist in the field. Recent technological advances, however, allow more in-depth investigation of innate and adaptive immune responses. Currently, the role of inflammasomes and neutrophil extracellular traps in the immune response to trauma is under investigation [129,130]. Importantly, current research uses induced rodent models of trauma, which is informative but also fails to recapitulate the complexity of naturally occurring trauma and immune responses in diverse species [131]. The need for diagnostic and therapeutic interventions in trauma and other diseases coupled with a high level of translational failure has increased awareness of the need to optimize research design, including the ability of models to demonstrate clinical efficacy in the target species [132]. The significant influence of polytrauma and patient factors such as comorbidities, exposure history, age, timing, trained immunity, and sex are productive areas of research. Finally, consideration for and investment in mechanisms, informatics, and technology to move research investigations to reliable, sustainable, bedside POC application to patients is needed as significant biomarkers and innovative treatments are identified.

An improved understanding of veterinary trauma immunology has the potential to strengthen both veterinary medical management of trauma as well as translational applications. Investment in veterinary trauma research has the potential to identify similarities as well as alternate adaptations, which can generate novel ideas and treatment pathways. Indeed, a more comprehensive understanding of trauma immunology can enhance our ability to identify concerns early and provide precise, personalized care, which prevents detrimental outcomes associated with dysregulated immune responses [129].

## Conclusion

Trauma is a common cause of morbidity and mortality in humans and companion animals. Recent efforts in procedural development, training, quality systems, data collection, and research have positively impacted patient outcomes; however, significant unmet need still exists. Coordinated efforts by collaborative, translational, multidisciplinary teams to advance trauma care and improve outcomes have the potential to benefit both human and veterinary patient populations and improve research sustainability. Strategic use of well-designed veterinary clinical trials informed by expertise along the research spectrum (i.e., benchtop discovery, comparative physiology, applied science and engineering, large laboratory animal models, clinical veterinary studies, and human randomized trials) can lead to increased therapeutic options for pets while accelerating and enhancing translation by providing early data to reduce the cost and the risk of failed human clinical trials.

Funding gaps remain a significant barrier to exploring integrated veterinary and human clinical development programs. Federal policy and funding priorities contribute to the gap, as veterinary research has restricted funding streams, is reviewed by a different population of reviewers, and is considered a separate field of study, despite similarities in biology, social and environmental factors, and injury patterns. Mechanisms to improve funding and support for integrated, innovative team science can accelerate needed, sustainable, and impactful progress in the care of major trauma.

## Supporting information

Hall et al. supplementary material 1 Hall et al. supplementary material

Hall et al. supplementary material 2 Hall et al. supplementary material

## Supplementary material

The supplementary material for this article can be found at https://doi.org/10.1017/cts.2024.513.
