# Supplementary material for: Breaking barriers in trauma research: A narrative review of opportunities to leverage veterinary trauma for accelerated translation to clinical solutions for pets and people
Source: J Clin Transl Sci. 2024 Apr 5;8(1):e74. doi: 10.1017/cts.2024.513 (PMC11075112; doi:10.1017/cts.2024.513)
Supplement: Hall et al. supplementary material 1 — Hall et al. supplementary material [file S2059866124005132sup001.docx]

Supplemental Table 1: A summary of advantages, disadvantages and resources available regarding various trauma models leveraged in the translational effort to advance trauma patient care and outcomes [human clinical, human volunteer, veterinary clinical, pre-clinical (induced animal models) and other]

|  | **MODELS AND RESOURCES FOR INTEGRATED SOLUTIONS** | | | | | |
| --- | --- | --- | --- | --- | --- | --- |
|  |  | **Human Clinical** | **Human Volunteer** | **Veterinary Clinical** | **Pre-clinical** | **Other (*in vitro*, *in silico,* etc.)** |
| **Resuscitation of the haemorrhaging patient** | **Advantages** | Species of interest | Species of interest: No cross-species confounders  Studies of relevant co-morbidities possible  ^1^ | Similar demographics, mechanisms of injury^2–5^  Similar pathophysiological responses^6,7^  Resource-rich hospitals with specialists, blood banks, and trauma focus^8^ | Range of trauma severities possible  Homogenous insults (results with fewer animals)  Pathophysiological response to haemorrhage similar across a range of species. ^9,10^ | Minimal if any ethical challenges / constraints  Cheap  High throughput |
|  | **Disadvantages** | Heterogeneity of injuries (may need large numbers for clinically meaningful results)  Consent  Co-morbidities  Which outcomes?^11^ | Must be no long-lasting effects: modelling mild haemorrhage only.  Lower body negative pressure does not fully model concurrent effects of tissue injury seen in trauma. | Species differences may limit translation  Different clinical practices impacting outcomes  Welfare concerns and use of euthanasia  Consent | Species differences may limit translation  Ethical challenges frequently limit studies to acute, anaesthetised, non-recovery models (long-term outcomes not assessed)  Rat as a trauma translational model^12^ | Response to haemorrhage involves multiple body systems and not possible to re-create all aspects *in vitro*  Validation required  Clarity on limitations/boundaries required |
|  | **Resources available** | Trauma network  databases^13,14^  Evidence-based Clinical Practice Guidelines^15,16^ | Lower body negative pressure (LBNP) to simulate physiological effects of haemorrhage^17,18^ | VetCOT trauma registry^19^  Future/underway: Clinical Practice Guidelines | Many research laboratories worldwide utilizing various species^20–22^ | Model of endotheliopathy^23^    Mathematical models of haemorrhagic shock^24^ |
| **Trauma -induced coagulopathy (TIC)** | **Advantages** | Species of interest | Species of interest: No cross-species confounders  Studies of relevant co-morbidities possible | Incidence is similar (approx. one third of moderate/severely injured in dogs) | Use of anaesthetised animal models enables replication of severe injury | Minimal if any ethical challenges / constraints  Cheap  High throughput |
|  | **Disadvantages** | Improvements in trauma care, early use of ‘blood’ and TXA for example  Prevalence (defined by laboratory tests) is lower as demonstrated in the ITACTIC study^8^ | Most prevalent in severe injury therefore modelling in volunteers not a viable option | Evidence of breadth of manifestations needs to be further defined  Focus of multi-center projects | Species differences in coagulation factor levels and laboratory test values as well as the relative contributions of fibrinogen and platelets exist; exact translation of temporal changes in the different species is deficient | The complex interaction between systems is difficult to replicate and validate |
|  | **Resources available** | Resuscitation protocols guided by viscoelastic testing^25,26^  Evidence-based Clinical Practice Guidelines^15,16,27^ | Acute hypercoagulation has been observed following LBNP^17^ | Similar clinical tools (viscoelastic testing)^28^ | Reviews^29–34^ | *Ex vivo* model^35–37^  State of the science review^38^ |
| **Traumatic brain Injury (TBI)** | **Advantages** | Species of interest | N/A | Similar range of mechanisms of injury  Similar validated scoring systems (MGCS) | A variety of models available with different mechanisms of injury^39^  Able to control severity of injury  Use of genetically engineered species to elucidate mechanisms^40^ | Minimal if any ethical challenges / constraints  Cheap  High throughput  Mechanistic studies |
|  | **Disadvantages** | Heterogeneity of injuries (may need large numbers for clinically meaningful results)  Consent  Onset of symptoms / progression of disease  Clinical meaningful outcomes? | N/A | Natural disease less well-characterised  Long-term effects not characterised  Less cognitive needs so may be better able to cope with enduring disability | Species variation in anatomy (e.g. lissencephalic and gyrencephalic brains)  Clinically meaningful outcomes can be difficult to replicate in animal models  Poor translation of therapeutics from animal models to human TBI patients^41^ | Current systems are deficient in many areas (e.g. *in vivo* microenvironment)^42^ |
|  | **Resources available** | Evidence-based Clinical Practice Guidelines^15,43^ |  |  | Animal model reviews^44^  Large animal model review^45,46^  Animal and non-animal models review^47^  Diagnostics/prognostics^48^ | Systematic review of *in vitro* models of TBI^49^  Non-mammal models of TBI^50,51^  *In vitro* and *ex vivo* models of TBI^52^  Review of computational models of TBI^53^ |
| **Translational Systems Biology** | **Advantages** | Biobanks / data repositories available for interrogation | Species of interest: No cross-species confounders  Studies of relevant co-morbidities possible | Similar demographics and mechanisms of injury | Wide range of models available for sample collection and  biobanking of samples feasible | Potential for a large amount of data generated from animal models that could be interrogated *in silico*  Modelling ‘cytokine storm’  Increasing field due to COVID-19 with potential opportunities to leverage models for trauma |
|  | **Disadvantages** | N/A | Most prevalent in severe injury therefore modelling in volunteers not a likely option | Currently a poorly studied field  Validity of companion animals as a model of post-trauma ‘omics unknown  ‘Self-selection’ (most severely injured die ‘pre-hospital’) | Long-term outcomes usually not assessed so translation may be limited |  |
|  | **Resources available** | Human studies^54–58^ |  |  | Mouse models^59,60^  Porcine model^61^  Rat model^62^ |  |
| **Trauma Immunology** | **Advantages** | Species of interest | Species of interest: No cross-species confounders  Studies of relevant co-morbidities possible | Similar demographics and mechanisms of injury for translation  Studies of relevant exposures and co-morbidities possible | Pre-injury status known |  |
|  | **Disadvantages** | Heterogeneity (age, sex, exposure, and genetic impacts; may need large numbers for clinically meaningful results) | Only observations in mild injury will be possible  Heterogeneity (age, sex, exposure, and genetic impacts; may need large numbers for clinically meaningful results) | Limited data available to understand trauma immunology in companion animals  Limited availability of suitable reagents  Heterogeneity (age, sex, exposure, and genetic impacts; may need large numbers for clinically meaningful results) | Limited volume of blood available in small mammals for longitudinal analysis  Limited availability of suitable reagents especially for large animal trauma models  Effects of stress and decreased immune exposures related to research environment | The complexity of the immune response is difficult to replicate *in vitro* |
|  | **Resources available** | Review^56^ | Experimental endotoxemia as a model of trauma^63^ |  | Review of animal models^64^ |  |

1. Lower Body Negative Pressure: Physiological Effects, Applications, and Implementation | Physiological Reviews. Accessed March 15, 2023. https://journals.physiology.org/doi/full/10.1152/physrev.00006.2018

2. Eastridge BJ, Holcomb JB, Shackelford S. Outcomes of traumatic hemorrhagic shock and the epidemiology of preventable death from injury. *Transfusion*. 2019;59(S2):1423-1428. doi:10.1111/trf.15161

3. Lee JA, Huang CM, Hall KE. Epidemiology of severe trauma in cats: An ACVECC VetCOT registry study. *J Vet Emerg Crit Care (San Antonio)*. 2022;32(6):705-713. doi:10.1111/vec.13229

4. Davros AM, Gregory CW, Cockrell DM, Hall KE. Comparison of clinical outcomes in cases of blunt, penetrating, and combination trauma in dogs: A VetCOT registry study. *J Vet Emerg Crit Care (San Antonio)*. 2023;33(1):74-80. doi:10.1111/vec.13253

5. Sise RG, Calvo RY, Spain DA, Weiser TG, Staudenmayer KL. The epidemiology of trauma-related mortality in the United States from 2002 to 2010. *J Trauma Acute Care Surg*. 2014;76(4):913-919; discussion 920. doi:10.1097/TA.0000000000000169

6. Hall K, Drobatz K. Volume Resuscitation in the Acutely Hemorrhaging Patient: Historic Use to Current Applications. *Front Vet Sci*. 2021;8:638104. doi:10.3389/fvets.2021.638104

7. Edwards TH, Rizzo JA, Pusateri AE. Hemorrhagic shock and hemostatic resuscitation in canine trauma. *Transfusion*. 2021;61(S1):S264-S274. doi:10.1111/trf.16516

8. VECCS | Facility Certification. Accessed March 6, 2023. https://veccs.org/facility-certification/

9. Tremoleda JL, Watts SA, Reynolds PS, Thiemermann C, Brohi K. Modeling Acute Traumatic Hemorrhagic Shock Injury: Challenges and Guidelines for Preclinical Studies. *Shock*. 2017;48(6):610. doi:10.1097/SHK.0000000000000901

10. Lomas-Niera JL, Perl M, Chung CS, Ayala A. SHOCK AND HEMORRHAGE: AN OVERVIEW OF ANIMAL MODELS. *Shock*. 2005;24:33. doi:10.1097/01.shk.0000191411.48719.ab

11. Holcomb JB, Moore EE, Sperry JL, et al. Evidence-Based and Clinically Relevant Outcomes for Hemorrhage Control Trauma Trials. *Annals of Surgery*. 2021;273(3):395. doi:10.1097/SLA.0000000000004563

12. Weber B, Lackner I, Haffner-Luntzer M, et al. Modeling trauma in rats: similarities to humans and potential pitfalls to consider. *Journal of Translational Medicine*. 2019;17(1):305. doi:10.1186/s12967-019-2052-7

13. TARN - Home. Accessed March 15, 2023. https://www.tarn.ac.uk/

14. National Trauma Data Bank® (NTDB®) | ACS. Accessed November 7, 2022. https://www.facs.org/quality-programs/trauma/quality/national-trauma-data-bank/

15. NICE | The National Institute for Health and Care Excellence. NICE. Accessed March 15, 2023. https://www.nice.org.uk/

16. Spahn DR, Bouillon B, Cerny V, et al. The European guideline on management of major bleeding and coagulopathy following trauma: fifth edition. *Critical Care*. 2019;23(1):98. doi:10.1186/s13054-019-2347-3

17. Cvirn G, Waha JE, Brix B, et al. Coagulation changes induced by lower-body negative pressure in men and women. *Journal of Applied Physiology*. 2019;126(5):1214-1222. doi:10.1152/japplphysiol.00940.2018

18. Hutchings SD, Watchorn J, McDonald R, et al. Quantification of stroke volume in a simulated healthy volunteer model of traumatic haemorrhage; a comparison of two non-invasive monitoring devices using error grid analysis alongside traditional measures of agreement. *PLOS ONE*. 2021;16(12):e0261546. doi:10.1371/journal.pone.0261546

19. Hall K. VetCOT: The Veterinary Trauma Registry. *Topics in Companion Animal Medicine*. 2019;37:100365. doi:10.1016/j.tcam.2019.100365

20. Hauser CJ. PRECLINICAL MODELS OF TRAUMATIC, HEMORRHAGIC SHOCK. *Shock*. 2005;24:24. doi:10.1097/01.shk.0000191387.18818.43

21. Hildebrand F, Andruszkow H, Huber-Lang M, Pape HC, van Griensven M. Combined Hemorrhage/Trauma Models in Pigs—Current State and Future Perspectives. *Shock*. 2013;40(4):247. doi:10.1097/SHK.0b013e3182a3cd74

22. Majde JA. Animal Models for Hemorrhage and Resuscitation Research. *Journal of Trauma and Acute Care Surgery*. 2003;54(5):S100. doi:10.1097/01.TA.0000064503.24416.F4

23. Pati S, Potter DR, Baimukanova G, Farrel DH, Holcomb JB, Schreiber MA. Modulating the endotheliopathy of trauma: Factor concentrate versus fresh frozen plasma. *J Trauma Acute Care Surg*. 2016;80(4):576-584; discussion 584-585. doi:10.1097/TA.0000000000000961

24. Curcio L, D’Orsi L, De Gaetano A. Seven Mathematical Models of Hemorrhagic Shock. *Computational and Mathematical Methods in Medicine*. 2021;2021:e6640638. doi:10.1155/2021/6640638

25. Baksaas-Aasen K, Gall LS, Stensballe J, et al. Viscoelastic haemostatic assay augmented protocols for major trauma haemorrhage (ITACTIC): a randomized, controlled trial. *Intensive Care Med*. 2021;47(1):49-59. doi:10.1007/s00134-020-06266-1

26. Lantry JH, Mason P, Logsdon MG, et al. Hemorrhagic Resuscitation Guided by Viscoelastography in Far-Forward Combat and Austere Civilian Environments: Goal-Directed Whole-Blood and Blood-Component Therapy Far from the Trauma Center. *Journal of Clinical Medicine*. 2022;11(2):356. doi:10.3390/jcm11020356

27. Damage control resuscitation in patients with severe traumatic hemorrhage - Practice Management Guideline. Accessed March 19, 2023. https://www.east.org/education-career-development/practice-management-guidelines/details/damage-control-resuscitation-in-patients-with-severe-traumatic-hemorrhage

28. Holowaychuk MK, Hanel RM, Darren Wood R, Rogers L, O’Keefe K, Monteith G. Prospective multicenter evaluation of coagulation abnormalities in dogs following severe acute trauma. *J Vet Emerg Crit Care (San Antonio)*. 2014;24(1):93-104. doi:10.1111/vec.12141

29. Ask A, Eltringham-Smith L, Bhakta V, Donkor DA, Pryzdial ELG, Sheffield WP. Spotlight on animal models of acute traumatic coagulopathy: an update. *Transfus Apher Sci*. 2022;61(2):103412. doi:10.1016/j.transci.2022.103412

30. van Zyl N, Reade MC, Fraser JF. Experimental Animal Models of Traumatic Coagulopathy: A Systematic Review. *Shock*. 2015;44(1):16. doi:10.1097/SHK.0000000000000372

31. Frith D, Cohen MJ, Brohi K. Animal models of trauma-induced coagulopathy. *Thromb Res*. 2012;129(5):551-556. doi:10.1016/j.thromres.2011.11.053

32. Stettler GR, Moore EE, Moore HB, et al. Thrombelastography Indicates Limitations of Animal Models of Trauma-Induced Coagulopathy. *J Surg Res*. 2017;217:207-212. doi:10.1016/j.jss.2017.05.027

33. Tarandovskiy ID, Shin HKH, Baek JH, Karnaukhova E, Buehler PW. Interspecies comparison of simultaneous thrombin and plasmin generation. *Scientific Reports*. 2020;10. doi:10.1038/s41598-020-60436-1

34. Gentry PA. Comparative aspects of blood coagulation. *Vet J*. 2004;168(3):238-251. doi:10.1016/j.tvjl.2003.09.013

35. Shenkman B, Budnik I, Einav Y, Hauschner H, Andrejchin M, Martinowitz U. Model of trauma-induced coagulopathy including hemodilution, fibrinolysis, acidosis, and hypothermia: Impact on blood coagulation and platelet function. *Journal of Trauma and Acute Care Surgery*. 2017;82(2):287. doi:10.1097/TA.0000000000001282

36. Li R, Elmongy H, Sims C, Diamond SL. Ex vivo recapitulation of trauma-induced coagulopathy and preliminary assessment of trauma patient platelet function under flow using microfluidic technology. *Journal of Trauma and Acute Care Surgery*. 2016;80(3):440. doi:10.1097/TA.0000000000000915

37. Caspers M, Schäfer N, Fröhlich M, et al. How do external factors contribute to the hypocoagulative state in trauma-induced coagulopathy? – In vitro analysis of the lethal triad in trauma. *Scand J Trauma Resusc Emerg Med*. 2018;26:66. doi:10.1186/s13049-018-0536-8

38. Tsiklidis E, Sims C, Sinno T, Diamond SL. Multiscale systems biology of Trauma Induced Coagulopathy. *Wiley Interdiscip Rev Syst Biol Med*. 2018;10(4):e1418. doi:10.1002/wsbm.1418

39. Ackermans NL, Varghese M, Wicinski B, et al. Unconventional animal models for traumatic brain injury and chronic traumatic encephalopathy. *Journal of Neuroscience Research*. 2021;99(10):2463-2477. doi:10.1002/jnr.24920

40. Traumatic brain injury and gene knockout animal models: an up-to-date review - Journal of Neurosurgical Sciences 2017 December;61(6):652-64. Accessed March 15, 2023. https://www.minervamedica.it/en/journals/neurosurgical-sciences/article.php?cod=R38Y2017N06A0652

41. Xiong Y, Mahmood A, Chopp M. Animal models of traumatic brain injury. *Nat Rev Neurosci*. 2013;14(2):128-142. doi:10.1038/nrn3407

42. Omelchenko A, Singh NK, Firestein BL. Current advances in in vitro models of central nervous system trauma. *Curr Opin Biomed Eng*. 2020;14:34-41. doi:10.1016/j.cobme.2020.05.002

43. Marshall S, Bayley M, McCullagh S, Velikonja D, Berrigan L. Clinical practice guidelines for mild traumatic brain injury and persistent symptoms. *Can Fam Physician*. 2012;58(3):257-267.

44. Petersen A, Soderstrom M, Saha B, Sharma P. Animal models of traumatic brain injury: a review of pathophysiology to biomarkers and treatments. *Exp Brain Res*. 2021;239(10):2939-2950. doi:10.1007/s00221-021-06178-6

45. Vink R. Large animal models of traumatic brain injury. *Journal of Neuroscience Research*. 2018;96(4):527-535. doi:10.1002/jnr.24079

46. Mayer AR, Dodd AB, Vermillion MS, et al. A systematic review of large animal models of combined traumatic brain injury and hemorrhagic shock. *Neurosci Biobehav Rev*. 2019;104:160-177. doi:10.1016/j.neubiorev.2019.06.024

47. Estrada-Rojo F, Martínez-Tapia RJ, Estrada-Bernal F, et al. Models used in the study of traumatic brain injury. *Reviews in the Neurosciences*. 2018;29(2):139-149. doi:10.1515/revneuro-2017-0028

48. Hajiaghamemar M, Seidi M, Oeur RA, Margulies SS. Toward development of clinically translatable diagnostic and prognostic metrics of traumatic brain injury using animal models: A review and a look forward. *Exp Neurol*. 2019;318:101-123. doi:10.1016/j.expneurol.2019.04.019

49. Wu YH, Rosset S, Lee T rin, Dragunow M, Park T, Shim V. In Vitro Models of Traumatic Brain Injury: A Systematic Review. *Journal of Neurotrauma*. 2021;38(17):2336-2372. doi:10.1089/neu.2020.7402

50. The utilization of small non‐mammals in traumatic brain injury research: A systematic review - PMC. Accessed March 15, 2023. https://www.ncbi.nlm.nih.gov/pmc/articles/PMC7941175/

51. Aggarwal P, Thapliyal D, Sarkar S. The past and present of Drosophila models of traumatic brain injury. *J Neurosci Methods*. 2022;371:109533. doi:10.1016/j.jneumeth.2022.109533

52. Hamilton KA, Santhakumar V. Current ex Vivo and in Vitro Approaches to Uncovering Mechanisms of Neurological Dysfunction after Traumatic Brain Injury. *Curr Opin Biomed Eng*. 2020;14:18-24. doi:10.1016/j.cobme.2020.05.001

53. Madhukar A, Ostoja-Starzewski M. Finite Element Methods in Human Head Impact Simulations: A Review. *Ann Biomed Eng*. 2019;47(9):1832-1854. doi:10.1007/s10439-019-02205-4

54. Thompson KB, Krispinsky LT, Stark RJ. Late immune consequences of combat trauma: a review of trauma-related immune dysfunction and potential therapies. *Mil Med Res*. 2019;6:11. doi:10.1186/s40779-019-0202-0

55. Wu J, Vodovotz Y, Abdelhamid S, et al. Multi-omic analysis in injured humans: Patterns align with outcomes and treatment responses. *Cell Rep Med*. 2021;2(12):100478. doi:10.1016/j.xcrm.2021.100478

56. Lord JM, Midwinter MJ, Chen YF, et al. The systemic immune response to trauma: an overview of pathophysiology and treatment. *Lancet*. 2014;384(9952):1455-1465. doi:10.1016/S0140-6736(14)60687-5

57. Cabrera CP, Manson J, Shepherd JM, et al. Signatures of inflammation and impending multiple organ dysfunction in the hyperacute phase of trauma: A prospective cohort study. *PLoS Med*. 2017;14(7):e1002352. doi:10.1371/journal.pmed.1002352

58. Sillesen M, Li Y, Alam HB. Transfusion Strategies are Associated with Epigenetic Changes Following Blunt Trauma. *Shock*. 2018;50(1):24-30. doi:10.1097/SHK.0000000000001035

59. Gihring A, Gärtner F, Schirmer M, Wabitsch M, Knippschild U. Recent Developments in Mouse Trauma Research Models: A Mini-Review. *Front Physiol*. 2022;13:866617. doi:10.3389/fphys.2022.866617

60. Relja B, Yang B, Bundkirchen K, Xu B, Köhler K, Neunaber C. Different experimental multiple trauma models induce comparable inflammation and organ injury. *Scientific Reports*. 2020;10. doi:10.1038/s41598-020-76499-z

61. Laserna AKC, Lai Y, Fang G, et al. Metabolic Profiling of a Porcine Combat Trauma-Injury Model Using NMR and Multi-Mode LC-MS Metabolomics—A Preliminary Study. *Metabolites*. 2020;10(9):373. doi:10.3390/metabo10090373

62. Slaughter AL, Nunns GR, D’Alessandro A, et al. The metabolopathy of tissue injury, hemorrhagic shock and resuscitation in a rat model. *Shock*. 2018;49(5):580-590. doi:10.1097/SHK.0000000000000948

63. Visser T, Pillay J, Pickkers P, Leenen LPH, Koenderman L. Homology in Systemic Neutrophil Response Induced by Human Experimental Endotoxemia and by Trauma. *Shock*. 2012;37(2):145. doi:10.1097/SHK.0b013e31823f14a4

64. Skelton JK, Purcell R. Preclinical models for studying immune responses to traumatic injury. *Immunology*. 2021;162(4):377-388. doi:10.1111/imm.13272
