# Supplementary material for: Breaking barriers in trauma research: A narrative review of opportunities to leverage veterinary trauma for accelerated translation to clinical solutions for pets and people
Source: J Clin Transl Sci. 2024 Apr 5;8(1):e74. doi: 10.1017/cts.2024.513 (PMC11075112; doi:10.1017/cts.2024.513)
Supplement: Hall et al. supplementary material 2 — Hall et al. supplementary material [file S2059866124005132sup002.docx]

Supplement 2: A list of veterinary trauma resources including regional Veterinary Trauma Centers (VTCs) and their level of verification. Like their human trauma center counter parts, VTCs are verified based on their depth of resources. The “Resources for the optimal care of the injured veterinary patient” document utilized to verify VTCs at each level described in the summary below can be accessed through the Veterinary Committee on Trauma (VetCOT) website under “Additional Resources”.

| **Veterinary Trauma Center** | **Location** | **Level** |
| --- | --- | --- |
| Animal Medical Center | New York, New York, USA | I |
| Arizona Veterinary Emergency and Critical Care Center | Peoria and Gilbert, Arizona, USA | III |
| Boundary Bay Veterinary Specialty Hospital | Bellingham, Washington, USA and Surrey, British Columbia, Canada | II |
| Canada West Veterinary Specialists and Critical Care Hospital | Vancouver, British Columbia, Canada | II |
| Colorado State University | Fort Collins, Colorado, USA | II |
| Iowa State University | Ames, Iowa, USA | II |
| Lakeshore Veterinary Specialists/Blue Pearl Hospital | Glendale, Wisconsin, USA | II |
| Maine Veterinary Medical Center | Scarborough, Maine, USA | II |
| North Carolina State University | Raleigh, North Caroline, USA | II |
| NorthStar VETS | Robbinsville, New Jersey, USA | II |
| Oakland Veterinary Referral Services | Bloomfield Hills, Michigan, USA | III |
| Ohio State University Veterinary Medical Center | Columbus, Ohio, USA | II |
| Oradell Animal Hospital | Paramus, New Jersey, USA | II |
| Pacific Northwest Pet ER and Specialty Center | Vancouver, Washington, USA | III |
| Red Bank Veterinary Hospital | Hillsborough, New Jersey, USA | I |
| The Royal Veterinary College, Queen Mother Hospital for Animals | London, United Kingdom | I |
| Tufts Cummings School of Veterinary Medicine | North Grafton, Massachusetts, USA | I |
| University of Pennsylvania School of Veterinary Medicine | Philadelphia, Pennsylvania, USA | I |
| Veterinary Specialty Center | Buffalo Grove, Illinois, USA | II |
| Veterinary Specialty Hospital of San Diego | San Diego, California, USA | II |
| Western Veterinary Specialty and Emergency Centre | Calgary, Alberta, Canada | II |
| Wisconsin Veterinary Referral Center | Grafton, Wisconsin, USA | II |

**Veterinary Trauma Center Levels^*^**

| **Level 1** - Level I VTCs must have the ability to provide total care for every aspect of management of the small animal trauma patient from emergency stabilization through definitive medical and surgical care, and rehabilitation. This requires significant depth of resources (medical and surgical) and personnel. These hospitals are open to receive SA trauma patients (and other emergencies) 24 hours a day, 7 days a week, 365 days a year. Level I VTCs also have the responsibility of providing leadership in education, training of veterinarians and veterinary technicians, and research. Level I VTCs are likely to have internship and/or residency programs in emergency and critical care (ECC); however, this is not a current requirement. Training for emergency veterinarians and technicians should involve, at a minimum, regular in-house continuing education specifically in the field of trauma. Level I VTCs have specialists available for in-house consultation 7-days a week in the fields of emergency and critical care, surgery, cardiology and radiology. In order to enable definitive care for trauma patients, specialists in anesthesiology, neurology, internal medicine, cardiology, and ophthalmology are also available on staff and on an on-call basis as needed. The intensive care unit (ICU) is under the supervision of an emergency and critical care specialist (DACVECC) and is staffed by experienced and/or certified veterinary technicians (CVTs) and veterinarians 24 hours a day, 7 days a week |
| --- |
| **Level II** - Level II VTCs are acute care facilities with the commitment, resources, and skilled personnel necessary to provide sophisticated medical and surgical, emergent, and critical care for critically ill or injured animals. They are expected to function as regional educational and/or research leaders, similar to Level I VTCs. These hospitals are open to receive SA trauma patients (and other emergencies) 24 hours a day, 7 days a week, 365 days a year. Level II VTCs must have specialists in emergency and critical care, surgery and internal medicine. Additionally these centers have a radiologist(s) available for consultation (if not available in-house). |
| **Level III** - Level III VTCs have the commitment, resources, and skilled personnel necessary to provide high level care for injured animals during business hours. These hospitals are open to receive SA trauma patients (and other emergencies) during part of the day but not necessarily 24 hours a day. Level III VTCs are primarily involved in the stabilization of severely traumatized patients and management of less severely injured trauma patients. There must be a veterinarian on duty, on the premises, at all times during hours of operation. These hospitals are not required to be staffed with veterinarians and technicians 24 hours a day and thus may transfer patients to a Level I or II VTC following initial stabilization, for ongoing care. Additionally, these hospitals are not required to have veterinarians with specialist qualifications on staff, and thus animals may be transferred to another facility for definitive medical or surgical care when necessary. Level III VTCs have procedures in place to allow consultation with, and easy transfer of patients to, Level I and/or II VTCs. |

^*^From: “Resources for the optimal care of the injured veterinary patient, Second edition”, JVECC 2018.

**Additional Resources**

American College of Veterinary Emergency and Critical Care – [www.acvecc.org](http://www.acvecc.org)

Clinical and Translational Science Award One Health Alliance – [www.ctdaonehealthalliance.org](http://www.ctdaonehealthalliance.org)

European College of Veterinary Emergency and Critical Care – [www.ecvecc.org](http://www.ecvecc.org)

Joint Trauma System, Department of Defense, USA – [jts.health.mil](file:///Users/wilkevets/Desktop/Kelly%20work/CSU/Research/TeTRA-Med/White%20paper%202022/Journal%20of%20Clinical%20and%20Translational%20Science%20submission/jts.health.mil)

Veterinary Committee on Trauma – [www.vetcot.org](http://www.vetcot.org)
